# Investigation of peri-implant tissue conditions and peri-implant tissue stability in implants placed with simultaneous augmentation procedure: a 3-year retrospective follow-up analysis of a newly developed bone level implant system

**DOI:** 10.1186/s40729-017-0104-4

**Published:** 2017-09-05

**Authors:** Jonas Lorenz, Henriette Lerner, Robert A. Sader, Shahram Ghanaati

**Affiliations:** 1 Department for Oral, FORM-Lab, Cranio-Maxillofacial, and Facial Plastic Surgery, Medical Center of the Goethe University Frankfurt, Frankfurt am Main, Germany; 2 HL-Dentclinic, Baden-Baden, Germany

**Keywords:** C-Tech implants, Guided bone regeneration, Oral implantology, Peri-implantitis

## Abstract

**Background:**

Guided bone regeneration (GBR) has been proven to be a reliable therapy to regenerate missing bone in cases of atrophy of the alveolar crest. The aim of the present retrospective analysis was to assess peri-implant tissue conditions and document peri-implant tissue stability in C-Tech implants when placed simultaneously with a GBR augmentation procedure.

**Methods:**

A total of 47 implants, which were placed simultaneously with a GBR procedure with a synthetic bone substitute material in 20 patients, were investigated clinically and radiologically at least 3 years after loading. Implant survival, the width and thickness of peri-implant keratinized gingiva, probing depth, bleeding on probing (BOP), the Pink Esthetic Score (PES), peri-implant bone loss, and the presence of peri-implant osteolysis were determined.

**Results:**

The follow-up investigation revealed a survival rate of 100% and only low median rates for probing depths (2.7 mm) and BOP (30%). The mean PES was 10.1 from the maximum value of 14. No osseous peri-implant defects were obvious, and the mean bone loss was 0.55 mm.

**Conclusions:**

In conclusion, implants placed in combination with a GBR procedure can achieve long-term stable functionally and esthetically satisfying results for replacing missing teeth in cases of atrophy of the alveolar crest.

## Background

The prevalence of peri-implantitis has grown in the past few years and has become a major issue in implant dentistry. Long-term stable and healthy soft- and hard-tissue conditions should be achieved in combination with esthetically and functionally satisfying results. However, the rising number of placed implants in the past decades has come with an increase in the prevalence of peri-implantitis [1].

Peri-implantitis is defined as a pathological inflammation of the peri-implant soft and hard tissue leading to peri-implant bone loss. For pathogenesis, many different factors are discussed in the literature. Reviews have shown that oral hygiene, implant surgery factors such as implant position, soft- and hard-tissue amount and quality, prosthetic concepts and design, general medical history, and other factors have an impact on the establishment and progression of peri-implantitis [2].

Peri-implant soft tissue forms the first border of the peri-implant tissue to the oral cavity and therefore to the migration of microorganisms that can cause and accelerate peri-implant infections. Dental implants, unlike the natural teeth, do not possess a compact barrier against penetration properties of the oral cavity. Peri-implant soft tissue acts as a cuff-like barrier [3]. In contrast to the periodontal attachment, there is no connective tissue fiber insertion into the implant surface. The peri-implant soft tissue possesses a lower number of blood vessels [4, 5] and cells but a higher amount of collagen [3, 6]. As a consequence of these anatomical differences, the peri-implant soft tissue has a decreased defending mechanism against microorganisms that in a pathological amount causes peri-implant infections.

A major etiological factor for peri-implantitis is the position of the implant in the surrounding bone [2]. In addition to bone quality and vascularization, a sufficient amount of peri-implant bone is important for the long-term stability of the implant and a sufficient underlining to the peri-implant soft tissue [2]. However, in most patients, the local bone amount is reduced due to atrophy, inflammatory processes, or resectional defects. Therefore, in the past few years, different techniques have been described to enlarge the local bone amount in prospective implant sites [7]. Besides methods such as GBR or the sinus augmentation technique, different augmentation materials have been investigated and established in the daily clinical routine. Autologous bone in the context of hard tissue augmentations is still the gold standard due to its osteogenic capacity [8]. To avoid the disadvantages that come with autologous bone transfer, such as a second surgical site and an increase in postoperative pain, biomaterial research has focused on the development of bone substitute materials that serve as scaffolds for the ingrowth of bone and its progenitor cells from the surrounding tissue [9].

The ability of bone substitute materials to form a sufficient and stable implantation bed has been proven in numerous clinical trials; however, it is still to a certain degree unclear if the different tissue reactions have an impact on the establishment of a peri-implant infection, especially when these biomaterials are used for augmentations around the implant shoulder. Due to the two-stage design of the implant, the implant shoulder presents a potential micro-gap between the abutment and the implant and a port of entry for microorganisms and peri-implant infections leading to a manifestation of peri-implantitis [10].

Regarding the stability of peri-implant hard and soft tissue, biological or anatomical factors are not the only elements that could be proven to have an impact. Technical factors such as the implant-abutment connection are also known to be key factors for long-term stable hard- and soft-tissue health [11]. Regarding the implant-abutment connection, which seems to be the key issue, located on the interface between the implant, the peri-implant bone, the peri-implant soft tissue, and the oral cavity, different studies have shown that a Morse-tapered conical connection reduces the micro-movement and therefore the micro-motions, which results in a pump effect of sulcus fluid and microorganisms in the fragile peri-implant soft tissue [10, 12]. The conical connection leads to a kind of “cold welding” type of connection that seems to prevent bone loss compared to external implant-abutment connections [10, 12].

A further factor, which has been detected to improve peri-implant hard- and soft-tissue health and is related to a conical implant-abutment connection, is a “platform switching” design. By switching the platform between the implant and the abutment from the outside surface of the implant to the inside region and therefore in larger distance to the peri-implant hard and soft tissue, the colonization of microorganisms seems to be reduced. Furthermore, the conical connection in combination with a platform switching design decreases stress transferred onto the peri-implant bone. As a result, peri-implant bone loss is prevented and the peri-implant soft- and hard-tissue health can be preserved [11, 13].

The aim of the present retrospective investigation was to assess clinically and radiologically peri-implant tissue conditions and document peri-implant tissue stability in C-Tech implants when placed simultaneously with a GBR augmentation procedure after at least 3 years of loading.

## Methods

### Patient population

In the present retrospective study, 47 dental implants (C-Tech Esthetic Line implants) from 20 patients (11 female, 9 male) with a mean age of 58.5 years (45–75 years) were analyzed clinically and radiologically. Implant placement and follow-up investigation was performed at the HL Dentclinic in Baden-Baden, Germany. The study was approved by the ethics commission of the medical department of Goethe University in Frankfurt am Main, Germany (378/16). All participating patients gave written informed consent to participate in the study and for publication of the obtained data. All patients from the private practice from one of the authors (H.L) that received C-Tech Esthetic Line implants in combination with a GBR augmentation procedure over a period of 1 year that have been available for follow-up investigation have been included in the present study. Furthermore, implants had to be loaded for at least 3 years. Patients with incomplete data collection or refusing to participate in the study have been excluded. Implants were placed in combination with simultaneous augmentation procedures on the implant shoulder (lateral augmentation, GBR) with synthetic (alloplastic) biomaterials. Hydroxyapatite (HA)-based bone substitute materials and bone substitute materials consisting of HA and beta-tricalcium phosphate (β-TCP) were used. Maxresorb^®^ (Botiss Biomaterials, Berlin, Germany) is a synthetic derived bone substitute material made of biphasic calcium phosphate. It is composed of 60% HA and 40% β-TCP and has been applied for augmentation in 26 implants, while in 21 implants, Osbone^®^ (Curasan, Frankfurt, Germany), a synthetic bone substitute material made of pure HA, has been used.

Implants were placed in native alveolar bone and augmentation around the implant shoulder due to horizontal and vertical bone defects that led to dehiscences of the implant surface. Twenty-three implants were placed in the upper jaw and 24 implants in the lower jaw. All implant placements were delayed at least 3 months after the extraction of teeth not worth preserving, and loading was done after a mean osseointegration period of 4 months. Prosthetic rehabilitation consisted of fixed prosthetics in 43 implants and removable prosthetics in 4 implants. The clinical and radiological follow-up investigation was performed after a loading period of at least 3 years (36–48 months after incorporation of prosthetics, mean 42.6 months). Implant survival and peri-implant hard- and soft-tissue health were analyzed to determine the manifestations of peri-mucositis by analysis of bleeding on probing (BOP) or peri-implantitis by analysis of marginal bone loss. Table 1 gives an overview of retrospectively investigated implants with patient information, implant localization, and implant data.

**Table 1:** Participating patients and the number and site of the inserted implants

Patient	Gender (m/f)	Age (years)	Implant localization (region)	Implant diameter (mm)	Implant length (mm)	Augmentation material	Prosthetic rehabilitation
1	f	50	32	3.5	13	HA + β-TCP	r.p
			34	4.3	11	HA + β-TCP	r.p
			42	3.5	13	HA + β-TCP	r.p
			44	4.3	11	HA + β-TCP	r.p
2	m	61	36	3.5	11	HA + β-TCP	f.p.
			37	3.5	11	HA + β-TCP	f.p.
			46	3.5	11	HA + β-TCP	f.p.
			47	3.5	11	HA + β-TCP	f.p.
3	m	48	26	4.3	11	HA + β-TCP	f.p.
4	f	54	21	4.3	11	HA + β-TCP	f.p.
5	f	45	23	3.5	13	HA	f.p.
			26	4.3	11	HA	f.p.
			27	4.3	11	HA	f.p.
6	m	56	32	3.5	13	HA + β-TCP	f.p.
			42	3.5	13	HA + β-TCP	f.p.
7	m	54	36	4.3	11	HA + β-TCP	f.p.
			46	3.5	11	HA + β-TCP	f.p.
			36	4.3	11	HA + β-TCP	f.p.
8	f	73	16	3.5	11	HA + β-TCP	f.p.
			26	3.5	11	HA + β-TCP	f.p.
9	m	64	27	4.3	11	HA + β-TCP	f.p.
10	f	62	15	3.5	11	HA + β-TCP	f.p.
			16	3.5	11	HA + β-TCP	f.p.
			17	3.5	11	HA + β-TCP	f.p.
			24	3.5	11	HA + β-TCP	f.p.
			36	4.3	11	HA + β-TCP	f.p.
			46	3.5	11	HA + β-TCP	f.p.
11	f	75	35	3.5	11	HA + β-TCP	f.p.
			36	3.5	11	HA + β-TCP	f.p.
12	f	52	16	4.3	11	HA + β-TCP	f.p.
13	m	46	24	3.5	11	HA + β-TCP	f.p.
			25	3.5	11	HA + β-TCP	f.p.
			26	3.5	11	HA + β-TCP	f.p.
			46	4.3	11	HA + β-TCP	f.p.
14	f	66	36	3.5	11	HA + β-TCP	f.p.
			37	3.5	11	HA + β-TCP	f.p.
15	f	63	11	3.5	13	HA	f.p.
16	f	53	36	3.5	11	HA + β-TCP	f.p.
			46	3.5	11	HA + β-TCP	f.p.
			47	3.5	11	HA + β-TCP	f.p.
17	f	51	14	3.5	13	HA + β-TCP	f.p.
			15	3.5	13	HA + β-TCP	f.p.
18	m	60	27	4.3	11	HA + β-TCP	f.p.
			47	4.3	11	HA + β-TCP	f.p.
19	m	75	22	3.5	13	HA + β-TCP	f.p.
			24	3.5	13	HA + β-TCP	f.p.
20	m	62	26	4.3	11	HA + β-TCP	f.p.
Total 20	Total 11*f; 9*m	Mean 58.5	Total 47; 23*u.j, 24*l.j.	Total 32*3.5 mm, 15*4.3 mm	Total 37*11 mm, 10*13 mm	Total 43*HA + β-TCP, 4*HA	Total 43*f.p., 4*r.p

*f* female, *m* male, *f.p*. fixed prosthetics, *r.p.* removable prosthetics, *u.j.* upper jaw, *l.j.* lower jaw, *HA + β-TCP* synthetic biphasic bone substitute material composed of 60% HA and 40% β-TCP, *HA* synthetic bone substitute material made of pure HA

### C-Tech implant system

In the present retrospective study, bone level implants (C-Tech Esthetic Line implants) were investigated clinically and radiologically. The bone level implant system has a Morse-locking conical implant-abutment connection with platform switching and an indexing hex that allows subcrestal implant placement and aims to prevent peri-implant bone loss. The surface of the implant system is manufactured by grit-blasting and acid-etching. The macrostructure of the implant consists of a beveled shoulder and three different threading profiles changing along the length of the implant.

**Fig. 1:**
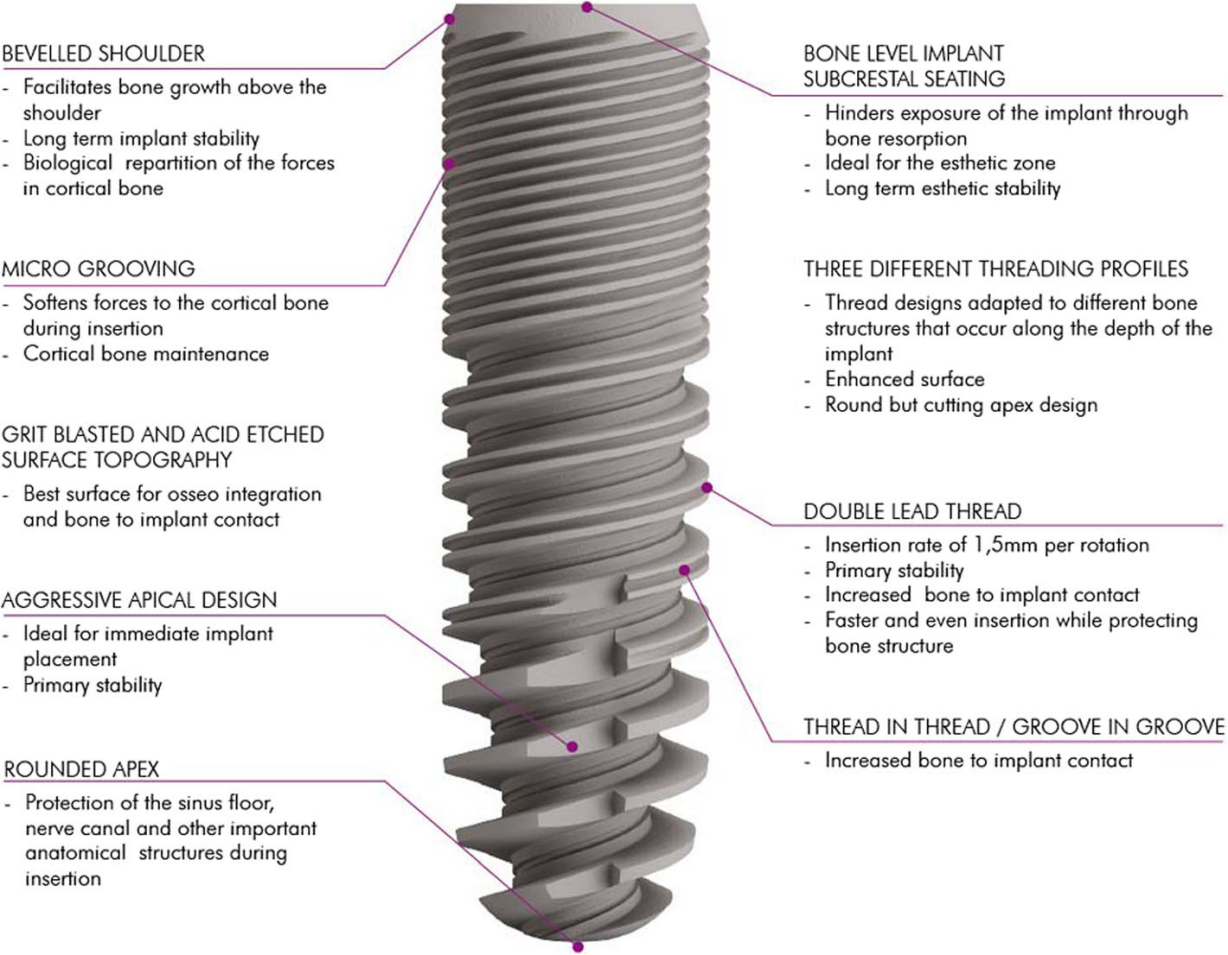
Schematic representation of the technical characteristics of the investigated C-Tech Esthetic Line implant system (provided by the manufacturer)

Figure 1 gives a representation of the technical characteristics of the investigated C-Tech Esthetic Line implant system.

### Clinical and radiological follow-up investigation

After a mean period of 3 years after loading, the implants were investigated clinically and radiologically according to previously published methods [14, 15]. To determine the stability of the peri-implant hard and soft tissue, the following parameters were analyzed: implant survival, that is, the implants being in situ; the width and thickness of the peri-implant keratinized gingiva (in millimeters); the probing depth (in millimeters); BOP; peri-implant bone loss (in millimeters); and the presence of peri-implant osteolysis. The probing depth was measured with a blunt periodontal probe at four sites (mesio-buccal, distal-buccal, mesio-oral, and disto-oral). Simultaneously to the measurement of the probing depths, the implant was checked to see if probing provoked bleeding (BOP).

To analyze the esthetic appearance of the implant-retained prosthetics, the PES was determined. Digital photographs including the neighboring and opposite teeth were recorded and evaluated by two independent experienced blinded investigators familiar with the PES scoring method. The PES score is generated using seven items (mesial papilla, distal papilla, soft-tissue level, soft-tissue contour, alveolar process deficiency, soft-tissue color, and texture) and an evaluation with a point score from 0 = very bad to 2 = excellent. Thus, a maximum score of 14 can be achieved. For determination of peri-implant bone loss, digitally recorded panoramic radiographies taken routinely after implant insertion and upon reexamination were analyzed with appropriate radiological software. Bone loss was measured mesially and distally, and a mean bone loss value was calculated.

Investigation parameters:

- Implant being in situ
- Width and thickness of peri-implant keratinized gingiva
- Pink Esthetic Score (PES)
- Probing depth
- BOP
- Peri-implant bone loss
- Presence of peri-implant osteolysis

## Results

Altogether, 47 implants were placed in the upper and lower jaws of a total of 20 patients. In all implants, lateral augmentation in a GBR process was performed simultaneously with implant placement due to reduced horizontal or vertical height of the alveolar crest. A total of 23 implants were placed in the upper jaw and 24 implants in the lower jaw. The implant diameter varied between 3.5 mm (32 implants) and 4.3 mm (15 implants). The implant length varied between 11 mm (37 implants) and 13 mm (10 implants). Prosthetic restoration consisted of fixed prosthetics (43 implants) and removable prosthetics (r.p.) (4 implants) (Table 1).

The bone substitute materials applied for the horizontal and vertical GBR procedures were of synthetic (HA and β-TCP) origin.

At the follow-up investigation 3 years after implant loading, all of the 47 placed implants were in situ, leading to a survival rate of 100%. No prosthetic complications, major infections, or incompatibility reactions were observed.

Clinical analysis of the probing depths and the presence of BOP was performed to uncover an inflammatory reaction in the peri-implant soft tissue. The mean probing depth calculated from the probing depths at four sites per implant was 2.4 mm, varying from 1 to 4 mm. BOP was observed during probing in 14 of the 47 implants (30%). A distinct correlation between an accumulation of increased probing depth and BOP was obvious, as most implants with BOP presented increased probing depths.

The amount of peri-implant attached keratinized gingiva in the implants of the present study was analyzed to determine a potential correlation between keratinized peri-implant gingiva, a potential inflammatory response, and peri-implant bone loss and peri-implant osteolysis. All implants had a band of keratinized gingiva of at least 1 mm width and thickness. The mean width was 3.2 mm, ranging from 2 to 6 mm, and the mean thickness was 2.4 mm, ranging from 1 to 4 mm. No distinct and statistically significant correlation of the amount of keratinized gingiva and the evaluated soft-tissue parameters (probing depth and BOP) was observed.

Investigation of the esthetic appearance via PES revealed a mean point score of 10.1 (ranging from 7 to 13) from a maximum of 14. The highest values and therefore acceptance were found in the alveolar process deficiency and the soft-tissue level, which can be interpreted as a benefit of the augmentation procedure around the implant shoulder.

Peri-implant bone loss calculated using the average bone loss mesially and distally of each implant was 0.55 mm (ranging from 0 to 3 mm) without any signs of acute infection or peri-implant osteolysis. Furthermore, the radiological analysis revealed a stable bone level in all implants 3 years after loading.

**Fig. 2:**
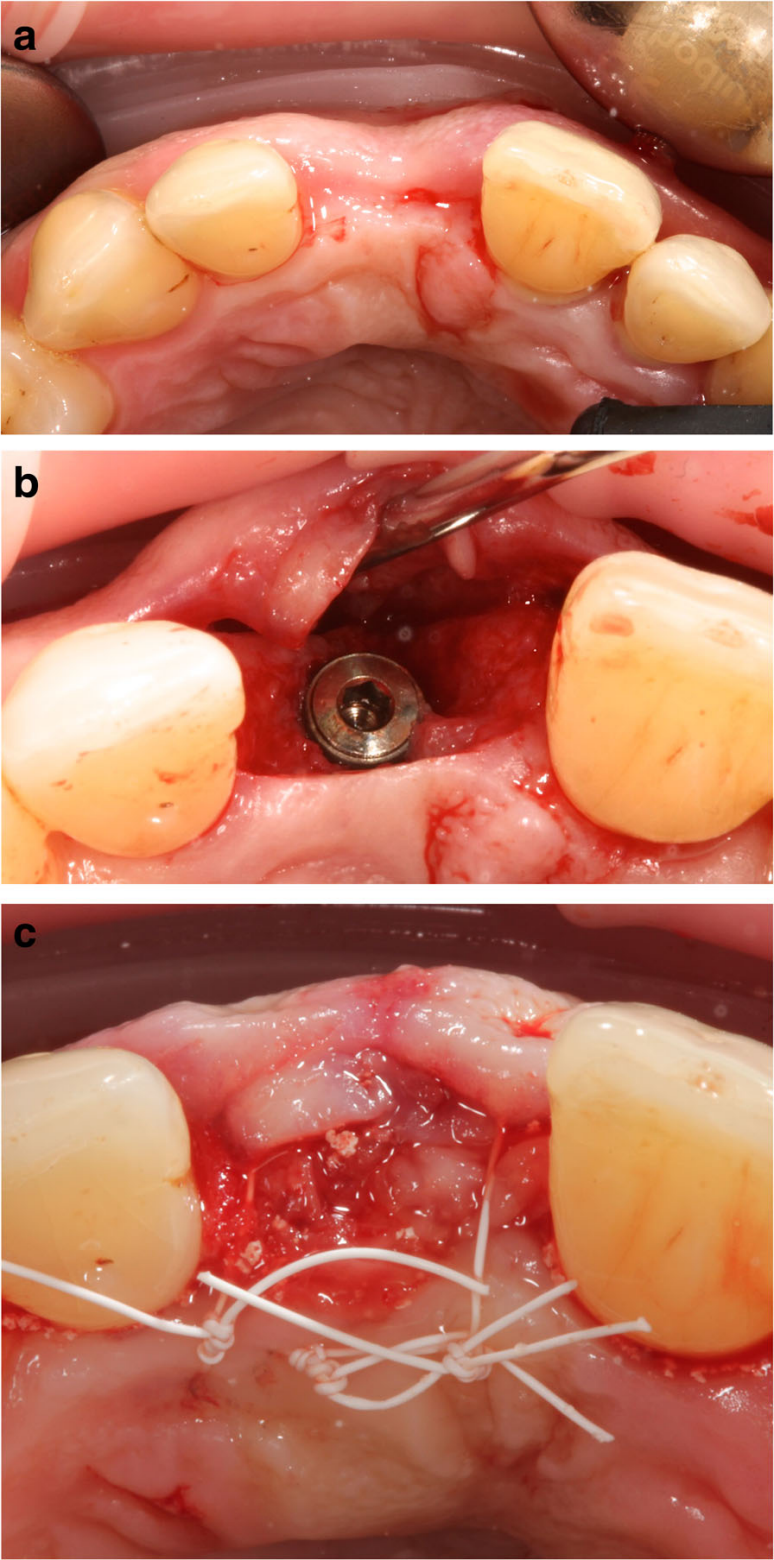
Clinical image of patient 4: **a** region 21 before implant placement. **b**, **c** Implant placement using the GBR procedure with a synthetic bone substitute material composed of HA + β-TCP

Table 2 gives an overview of the results of the clinical and radiological 3-year follow-up investigation. Figure 2a–d shows clinical images of the placed implant in patient 4.

**Table 2:** Results from the clinical and radiological 3-year follow-up investigation

Patient	Implant-localization (region)	Implant loss (+/−)	Buccal width of keratinized peri-implant gingiva (mm)	Buccal thickness of keratinized peri-implant gingiva (mm)	Pink Esthetic Score (PES)	Probing depth (mm) at four sites (mb, db, mo, do)	Bleeding on Probing (+/−) at four sites (mb, db, mo, do)	Peri-implant bone loss (mm)	Presence of peri-implant osteolysis (+/−)
1	32	**−**	2	2	**−**	3, 2, 2, 3	−, −, −, −	0	**−**
	34	**−**	3	3	**−**	2, 2, 2, 3	−, −, −, −	0	**−**
	42	**−**	3	2	**−**	3, 2, 3, 2	−, −, −, −	0	**−**
	44	**−**	2	3	**−**	3, 3, 2, 3	−, −, −, −	0	**−**
2	36	**−**	2	3	8	3, 3, 3, 4	−, −, −, +	0.5	**−**
	37	**−**	2	3	7	2, 3, 2, 3	−, −, −, −	0.5	**−**
	46	**−**	3	2	8	3, 3, 2, 3	−, −, −, −	0	**−**
	47	**−**	3	2	9	3, 4, 3, 4	−, +, −, +	0	**−**
3	26	**−**	4	3	8	2, 3, 3, 3	−, −, −, −	1	**−**
4	21	**−**	3	3	8	2, 2, 2, 3	−, −, −, −	0	**−**
5	23	**−**	4	2	9	3, 2, 2, 2	−, −, −, −	0	**−**
	26	**−**	3	2	9	3, 3, 3, 4	−, −, −, +	0.5	**−**
	27	**−**	3	3	8	3, 4, 4, 4	−, +, −, +	1	**−**
6	32	**−**	3	2	11	2, 3, 2, 3	−, −, −, −	1	**−**
	42	**−**	2	2	11	2, 1, 1, 2	−, −, −, −	0	**−**
7	36	**−**	3	2	10	3, 3, 3, 4	−, +, −, −	0.5	**−**
	46	**−**	2	3	9	4, 5, 3, 4	+, −, +, −	0.5	**−**
	36	**−**	3	3	10	3, 2, 2, 3	−, −, −, −	3	**−**
8	16	**−**	3	2	11	2, 2, 2, 3	−, −, −, −	0.5	**−**
	26	**−**	3	2	10	3, 2, 2, 2	−, −, −, −	1	**−**
9	27	**−**	3	2	9	3, 3, 3, 4	−, −, −, +	1	**−**
10	15	**−**	4	3	12	3, 2, 2, 2	−, −, −, −	0	**−**
	16	**−**	4	3	11	3, 3, 2, 2	−, −, −, −	0	**−**
	17	**−**	3	2	9	3, 3, 4, 3	−, −, +, −	0	**−**
	24	**−**	4	4	12	2, 3, 2, 3	−, −, −, −	0	**−**
	36	**−**	2	1	10	3, 4, 3, 3	−, +, −, −	0.5	**−**
	46	**−**	2	2	9	3, 3, 3, 3	−, −, −, −	1	**−**
11	35	**−**	3	2	11	2, 2, 3, 2	−, −, −, −	0.5	**−**
	36	**−**	3	2	11	3, 3, 2, 2	−, −, −, −	1	**−**
12	16	**−**	4	2	12	3, 2, 2, 2	−, −, −, −	0	**−**
13	24	**−**	5	3	12	1, 2, 2, 2	−, −, −, −	0	**−**
	25	**−**	5	2	11	2, 2, 1, 1	−, −, −, −	0	**−**
	26	**−**	4	2	9	2, 2, 3, 2	−, −,−, −	1	**−**
	46	**−**	3	2	8	3, 3, 4, 3	−, −, +, −	2	**−**
14	36	**−**	3	2	7	4, 3, 3, 2	+, −, −, −	0.5	**−**
	37	**−**	2	2	10	3, 4, 3, 3	−, −, −, −	1	**−**
15	11	**−**	4	3	13	2, 2, 3, 2	−, −, −, −	0	**−**
16	36	**−**	2	2	11	3, 3, 2, 2	−, −, −, −	0.5	**−**
	46	**−**	3	2	10	3, 4, 3, 3	−, +, −, −	1	**−**
	47	**−**	2	2	11	3, 3, 2, 3	−, −, −, −	1	**−**
17	14	**−**	5	2	12	2, 2, 1, 2	−, −, −, −	0	**−**
	15	**−**	4	3	13	2, 2, 2, 3	−, −, −, −	0	**−**
18	27	**−**	3	2	11	3, 3, 4, 3	−, +, −, +	1	**−**
	47	**−**	2	2	9	4, 4, 3, 3	+, −, −, −	2	**−**
19	22	**−**	5	3	13	2, 2, 1, 2	−, −, −, −	0	**−**
	24	**−**	6	3	12	2, 3, 3, 2	−, −, −, −	1	**−**
20	26	**−**	4	2	11	3, 3, 2, 3	−, −, −, −	1	**−**
Total 20	Total 47; 23*u.j, 24*l.j.	Total 0	Mean 3.2 mm (2–6 mm)	Mean 2.4 mm (1–4 mm)	Mean 10.1 (7–13)	Mean 2.7 mm (1–5 mm)	Total 9.6% of the sites; 30% of the implants	Mean 0.55 mm (0–3 mm)	Total 0

*mb* mesio-buccal, *db* disto-buccal, *mo* mesio-oral, *do* disto-oral, *+* present, *−* absent, *f.p.* fixed prosthetics, *r.p.* removable prosthetics, *u.j.* upper jaw, *l.j.* lower jaw

## Discussion

In the present retrospective study, C-Tech bone level implants placed simultaneously with a GBR procedure around the implant shoulder were investigated clinically and radiologically after at least 3 years of loading to assess peri-implant tissue conditions and document peri-implant tissue stability.

A total of 47 implants were placed in the upper (23 implants) and lower jaw (24 implants) of 20 patients. In all implants, lateral augmentation in a GBR process was performed simultaneously with implant placement due to a reduced horizontal or vertical height of the alveolar crest. The bone substitute materials applied to the horizontal and vertical GBR procedures were of synthetic origin. The clinical and radiological follow-up investigation revealed a survival rate of 100% and only low median rates for probing depths (2.7 mm) and BOP (30%). The mean PES was 10.1 from a maximum value of 14. No osseous peri-implant defects were obvious, and the mean bone loss calculated digitally was 0.55 mm, ranging from 0 to 3 mm.

The tissue reactions to bone substitute materials of different origins have been widely investigated by our research group [16–18]. It could be shown that the origin, the physico-chemical structure, and the processing techniques have an impact on the cellular tissue reaction within the augmentation bed. In a clinical study, the tissue reaction to a synthetic, HA-based and xenogeneic, bovine-based bone substitute material was compared histologically and histomorphometrically in a two-stage sinus augmentation procedure. It was shown that the synthetic bone substitute material induced a significantly higher expression of multinucleated giant cells (MNGCs) within the implantation bed compared to the xenogeneic bone substitute material. However, the induced MNGC-related tissue reaction came with a significantly higher vascularization within the implantation bed. Regarding the new bone formation within the implantation bed, it must be mentioned that the results of new bone formation after an integration period of 6 months did not differ between the synthetic and the xenogeneic bone substitute material [16].

The tissue reaction, however, did not only differ in bone substitute materials of different origin but also in bone substitute materials of the same origin. In an in vivo trial, two xenogeneic bone substitute materials processed with different techniques were implanted subcutaneously in CD-1 mice for up to 60 days. Both bone substitute materials showed good integration within the peri-implant tissue with no signs of adverse inflammatory effects. However, within the implantation bed of the bone substitute of low sintering temperature, few MNGCs were obvious on the surface of small bone substitute granules in the early integration period, while the tissue reaction to the larger granules at later stages consisted mainly of mononuclear cells. In contrast, the tissue reaction to the bone substitute material of high sintering temperature consisted mainly of biomaterial surface-associated MNGCs. Previous in vivo and clinical investigations indicated that MNGCs, which are widely known to be an expression of an ongoing foreign body reaction, are expressed especially on the surface of synthetic biomaterial granules, trying to degrade the biomaterial, but without really reducing the ratio of the biomaterial. In fact, multinucleated giant cells, which do not have the ability of degrading synthetic bone substitute materials, can be characterized more as foreign body giant cells than as osteoclastic cells [17–19].

Previously, our group performed another retrospective study with the same bone level implant system placed immediately after the extraction of teeth not worth preserving [14]. In a collective of 21 patients (11 female, 10 male), 50 dental implants were placed immediately in fresh extraction sockets in the upper (31 implants) and lower jaws (19 implants). The same clinical and radiological parameters were applied to investigate implants 2 years after loading. During the mean observation period of 2 years, none of the implants failed or presented an acute infection or peri-implantitis. All of the implants presented a sufficient amount of peri-implant keratinized soft tissue, low rates of probing depth (mean 2.25 mm), and presence of BOP (34%). The peri-implant bone level was stable, with a mean bone loss 2 years after loading of 0.83 mm [14].

Comparing the present results to the aforementioned study with the same implant system on immediately placed implants, it seems that the GBR augmentation procedure has no influence on the long-term stability of the implants. In both studies with different placement modalities and protocols, comparable clinical and radiological results were achieved. This leads to the assumption that the investigated C-Tech bone level implant system is able to achieve long-term stable function and to render esthetically satisfying results for replacing missing teeth in cases of atrophy of the alveolar crest, as well as in cases of immediate implant placement.

However, the biomaterial-related tissue reaction is still not clarified in detail and more studies need to be performed to investigate the interaction of biomaterials, such as bone substitute materials and dental implants.

## Conclusions

In the present study, the implant and peri-implant hard- and soft-tissue stability was analyzed in a bone level implant system placed simultaneously with a GBR procedure 3 years after prosthetic loading. Peri-implant hard- and soft-tissue parameters such as width and thickness of peri-implant keratinized gingiva, probing depth, BOP, PES, peri-implant bone loss, and the presence of peri-implant osteolysis were analyzed. The 3-year follow-up investigation revealed a survival rate of 100% and comparably low values for probing depth (2.7 mm) and BOP (30%). Furthermore, analysis of PES showed a favorable esthetic appearance of the implants and prosthetics. The synthetic HA and HA + β-tricalcium phosphate-based bone substitute materials used for the GBR seem to have had no negative influence on the peri-implant health, as all investigated parameters were in accordance with or better than the results presented in the international literature. In conclusion, the investigated bone level implant system seems to be suitable to achieve functionally and esthetically satisfying results in indications that require simultaneous augmentation procedures 3 years after loading.

## Abbreviations

β-TCP: β-tricalcium phosphate
BOP: Bleeding on probing
F.P.: Fixed prosthetics
GBR: Guided bone regeneration
HA: Hydroxyapatite
MNGCs: Multinucleated giant cells
PES: Pink Esthetic Score
R.P.: Removable prosthetics
